# The role of reinforcement learning and value-based decision-making frameworks in understanding food choice and eating behaviors

**DOI:** 10.3389/fnut.2022.1021868

**Published:** 2022-11-22

**Authors:** Alaina L. Pearce, Bari A. Fuchs, Kathleen L. Keller

**Affiliations:** ^1^Social Science Research Institute, Pennsylvania State University, University Park, PA, United States; ^2^Department of Nutritional Sciences, Pennsylvania State University, University Park, PA, United States; ^3^Department of Food Science, Pennsylvania State University, University Park, PA, United States

**Keywords:** food choice, obesity, value-based decision-making, reinforcement learning, model-free vs. model-based learning, sign-and goal-tracking

## Abstract

The obesogenic food environment includes easy access to highly-palatable, energy-dense, “ultra-processed” foods that are heavily marketed to consumers; therefore, it is critical to understand the neurocognitive processes the underlie overeating in response to environmental food-cues (e.g., food images, food branding/advertisements). Eating habits are learned through reinforcement, which is the process through which environmental food cues become valued and influence behavior. This process is supported by multiple behavioral control systems (e.g., Pavlovian, Habitual, Goal-Directed). Therefore, using neurocognitive frameworks for reinforcement learning and value-based decision-making can improve our understanding of food-choice and eating behaviors. Specifically, the role of reinforcement learning in eating behaviors was considered using the frameworks of (1) Sign-versus Goal-Tracking Phenotypes; (2) Model-Free versus Model-Based; and (3) the Utility or Value-Based Model. The sign-and goal-tracking phenotypes may contribute a mechanistic insight on the role of food-cue incentive salience in two prevailing models of overconsumption–the Extended Behavioral Susceptibility Theory and the Reactivity to Embedded Food Cues in Advertising Model. Similarly, the model-free versus model-based framework may contribute insight to the Extended Behavioral Susceptibility Theory and the Healthy Food Promotion Model. Finally, the value-based model provides a framework for understanding how all three learning systems are integrated to influence food choice. Together, these frameworks can provide mechanistic insight to existing models of food choice and overconsumption and may contribute to the development of future prevention and treatment efforts.

## Introduction

Each day we make hundreds of choices about what to eat, many of which occur automatically with little conscious thought (1). While in lay terms, the phrase “food choice” is often limited to the decisions about the composition of a meal (e.g., *What’s for dinner?*), the current review uses a broader definition that encompasses the behavioral and environmental factors that influence meal initiation, amount consumed, and quality of the food choices (2–4). Food choices are extremely complex because they evolve over varying time scales, have multiple determinants, and occur within various contexts (e.g., celebratory, meals, and snacks) (2, 3, 5). Adding to the complexity is the overwhelming influence of the obesogenic food environment, which makes highly palatable, energy-dense (i.e., “ultra-processed”) foods more affordable and accessible (6). Food choice in the context of an obesogenic environment requires the integration of multiple, often conflicting pieces of information (3). For example, presence of food cues such as McDonald’s “Golden Arches” may trigger wanting for energy-dense foods (e.g., Big Mac and French fries) that are not compatible with goals to maintain a healthy diet (7). With 1 in 5 deaths linked to a poor diet (8, 9) and obesity rates among children continuing to rise (10), it is critically important to understand how food choices are made in response to environmental food cues (e.g., food images, advertising/branding). Understanding the neurocognitive processes those underly food choices in this context is crucial for the development of effective, tailored health interventions.

Environmental food cues influence food choice through three behavioral controllers or systems: Pavlovian, instrumental/habit, and goal-directed (1, 11, 12). The Pavlovian system regulates automatic behavioral responses to cues that are associated with evolutionarily relevant outcomes. The classical example is of Pavlov’s dogs salivating at the sight of food (13). While these responses can be present without learning (i.e., “hard-wired”), the association between a stimulus or cue (e.g., bell sounding) and an evolutionarily significant outcome (e.g., food delivery) can be learned and presumably confers selective advantages to human and non-human animals in their search for edible and nutritious foods (11, 13, 14). For example, approaching a cue that predicts food delivery (1, 11) or consuming all the food available on a plate regardless of hunger would be considered Pavlovian behaviors (11). In contrast to the Pavlovian system where the outcome or reward is delivered regardless of behavior, in instrumental learning, reward delivery is contingent upon the behavior performed in response to the cue (11, 14, 15). Thus, while the Pavlovian system supports stimulus-outcome (S-O) learning the instrumental system supports stimulus-response (S-R) learning. The instrumental system has also been termed the “habit” system because learned actions can occur even when the outcome is not desired, which can lead to habitual behaviors (15). For example, the instrumental system would drive habitual coffee intake at a specific time of day regardless of whether the stimulating effect of caffeine is needed or desired (1, 11, 15). While a habitual behavior may occur regardless of state as in the prior example, the value of food-related actions is also influenced by internal states like hunger (16–18). In contrast to the instrumental system which is driven by previously learned S-R associations, the goal-directed system prospectively evaluates response-outcome (R-O) associations based on the anticipated or predicted outcome for each action (1, 11, 15). For example, the goal-directed behavior of choosing where to eat in a novel city would be driven by the anticipated value for the food at each restaurant. Together, these three systems drive eating behavior and food choice in response to environmental food cues.

While the instrumental and goal-directed systems contribute to value-based decision-making in general, food choice is a unique because it can also be influenced by the Pavlovian system (1). Therefore, applying neurocognitive frameworks to understanding the factors that motivate food choice may elucidate novel behaviors to target in dietary interventions. The current review is intended to provide an overview of three frameworks that encompass these learning systems: (1) sign-and goal-tracking phenotypes; (2) model-based and model-free reinforcement learning; and (3) the utility or value-based model. For each framework we will provide a brief translational review of the theory and its supporting neurobiological substrates, followed by a summary of possible applications to understanding food choice and eating behaviors. Finally, we will consider how these frameworks can be utilized to improve understanding of food-choice and applied to the development of more effective prevention/treatment programs for disordered or dysregulated eating.

## Sign-and goal-tracking

**FIGURE 1:**
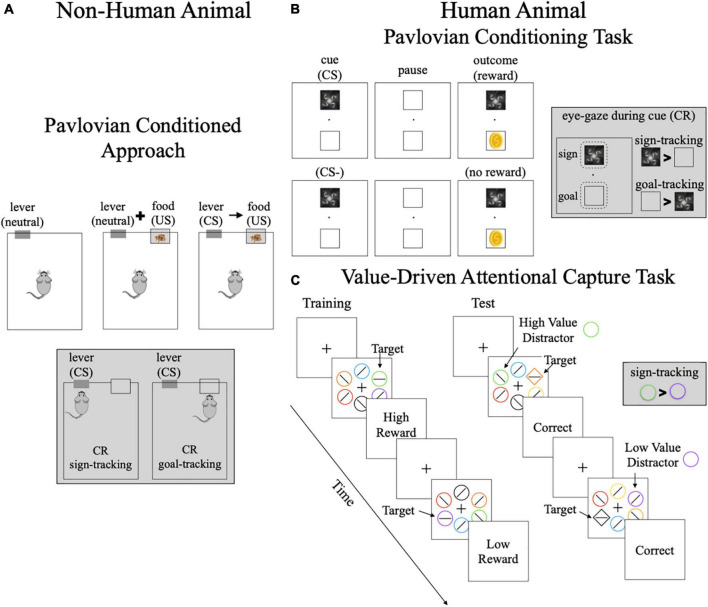
Methods of assessing sign-and goal-tracking phenotypes. **(A)** Pavlovian conditioned approach task–this task is used in non-human animal models. Animals learn the stimulus-outcome (S–O) association between the lever and food delivery. The conditioned responses are: (1) sign-trackers–approach the lever; (2) goal-trackers–approach the location of food delivery. **(B)** Pavlovian conditioning task–this is a simplified schematic of a Pavlovian conditioning task where a conditioned stimulus either predicts reward receipt or no reward. The conditioned stimulus and reward are presented in different locations so the conditioned response of eye-gaze can be measured. The conditioned responses are: (1) sign-trackers–look at the conditioned stimulus more than reward location; (2) goal-trackers–look more at the reward location than conditioned response. **(C)** Value-Driven Attentional Capture Task–this task includes a training and a test phase. Both phases include a visual search task where participants must locate the location of the horizontal line. During training, one color is associated with high reward (e.g., green) and one color is associated with low reward (e.g., purple). During the test phase, the target is the unique shape and the previously rewarded colors are used as high or low value distractors. No reward is given for correct responses in the test phase. The conditioned responses are: (1) sign-trackers–looking at the previously rewarded cue, resulting in slower reaction times; (2) goal-trackers–not distracted by previously rewarded cues. CS, conditioned stimuli; CR, conditioned response; US, unconditioned stimuli. Gray boxes highlight the definition of sign-and goal-tracking for each task.

The sign-and goal-tracking phenotype is an animal model for motivational control of behavior in response to environmental cues (19–23). These phenotypes are characterized in animals using the Pavlovian Conditioned Approach (PCA) test (24, 25). Pavlovian conditioning occurs when a neutral cue (e.g., lever) becomes a conditioned stimulus (CS) after being repeatedly paired with an unconditioned stimulus (US) like food. In the PCA test (Figure 1A), a lever (neutral) is repeatedly presented prior to food delivery (US) allowing the animal to learn the lever-food (S-O) association (Figure 1A). Once the lever becomes a CS, it is able to elicit conditioned responses (CR) (22, 24, 25). Animals display three patterns of CRs: (1) goal-tracking: approaching the location of food delivery (US); (2) sign-tracking: approaching the lever (CS) itself; and (3) intermediate: switching between the two CRs (20–22, 25). Importantly, all animals are equally able to learn the S-O association regardless of CR displayed (26). The differing patterns of CRs occur due to differences in the attribution of incentive salience or motivational value to the CS (20–22, 25). For sign-trackers, the CS becomes an incentivized stimulus, which has three defining properties: (1) it biases attention; (2) it is desired and the animal will work for it (i.e., is “wanted”); and (3) it can increase motivation to seek reward (20–22, 25, 27). Once the CS becomes desired, sign-trackers will approach and interact with the CS even if it means losing access to the primary reward (e.g., food) (25). Therefore, a key behavioral distinction between these phenotypes is the propensity for environmental cues to take on rewarding properties and motivate wanting.

### Neural pathways that support sign-and goal-tracking phenotypes

The sign-and goal-tracking phenotypes have well-characterized differences in neural engagement during stimulus-reward learning and attribution of incentive salience. Sign-trackers show greater phasic dopaminergic (DA) signaling in ventral striatum, a region integral in stimulus-reward learning, which has been linked to the attribution of incentive salience to the CS (21, 22, 26, 28, 29). Sign-trackers also show a higher firing rate for excitatory signals in response to the CS in ventral pallidum (30), a subcortical region that is important for motivated behaviors and incentive salience (31). While both ventral striatum and pallidum have “hedonic hotspots” that enhance hedonic influence of the CS (31–33), incentive motivation or wanting of the CS (i.e., sign-tracking) seems to be driven by projections from ventral striatum to ventral pallidum (34). Although sign-tracking seems to be driven by these subcortical DA-related signaling differences, there are also important differences in cortical signaling. In particular, sign-trackers show cortical differences in acetylcholine (ACh), a neuromodulator that is important for attentional control and learning. In response to attentional demands, sign-trackers are less able to upregulate ACh which leads to stimulus-driven or bottom-up attention control [for review see (24)]. Therefore, sign-trackers show a pattern of greater signaling in subcortical “hedonic hotspots” in conjunction with a reduced cortical ACh signaling, which limits engagement top-down attentional control.

The pattern of greater bottom-up reward signaling and reduced top-down control signaling in sign-compared to goal-trackers is paralleled by circuit-level differences. Cue-motivated behaviors driven by incentive salience involve widespread circuits including cortical, thalamic pallidum, and striatal loops that converge in the ventral striatum (21, 33, 35). Sign-trackers have greater engagement of ventral and dorsal striatum (i.e., caudate-putamen) during stimulus-reward learning while goal-trackers show greater engagement of prefrontal cortical regions [for review see (21)]. Therefore, it has been hypothesized that cue-motivated behaviors are subserved by subcortical circuits while top-down cortical circuits inhibit the attribution of incentive salience to cues (21). Reduced engagement of cortical regions associated with top-down control may also contribute to greater impulsivity (36, 37) and reduced behavioral flexibility (38) observed in sign-trackers compared to goal-trackers. Together, this suggests neural differences between phenotypes contribute to differences in attribution of incentive salience and may also be related to differences in attentional control and impulsivity (21, 24).

### Translation of sign-and goal-tracking phenotypes to humans

In humans, sign-and goal-tracking have been characterized using Pavlovian conditioning tasks (often as part of the Pavlovian instrumental transfer paradigm) and the Value-Drive Attentional Capture (VDAC) task. Using eye-tracking, incentive salience can be measured in Pavlovian conditioning tasks by examining the amount of time looking at the location of the CS compared to the location where reward is delivered (Figure 1B). Much like sign-tracking animals that fixate on the lever rather than location of food delivery, adult humans who spend more time looking at the location of the CS compared to the reward have also been classified as sign-trackers (39, 40). In line with the animal phenotype of sign-tracking, adults classified as sign-trackers during Pavlovian conditioning show greater impulsivity than those classified as goal-trackers (39). Similarly, VDAC tasks (Figure 1C) measure attentional bias toward high-value stimuli, however, these tasks assess this bias when the stimuli are no longer relevant to the task goal and are no longer rewarded (41–43). Continued attentional bias toward previous high-value stimuli–termed attentional capture–reflects the attribution of incentive salience to these stimuli (27, 43–45) and sign-tracking (27, 45). Greater attentional capture on the VDAC has been associated with greater compulsivity (45, 46) and impulsivity (41) as well as risk for substance use disorder (45). Together, this shows that behavioral profiles associated with sign-tracking have similarities in human and non-human animals (e.g., impulsivity, poor attentional control).

### Relevance to food choice and eating behaviors

The sign-and goal-tracking phenotype model has high translational potential to inform our understanding of food choice and overconsumption. This is supported by animal studies which have shown that obesity-prone rats display greater attribution of incentive salience compared to obesity-resistant models (47). There is initial evidence that obesity is associated with cue-outcome behavioral responses that are indicative of sign-tracking. In a Pavlovian conditioning task that paired visual cues with receipt of chocolate milkshake, water, or nothing, adults with overweight showed the CR of increased swallowing in response to cues that predicted chocolate milkshake delivery while adults with healthy weight did not (48). This suggests that adults with overweight were more likely to attribute incentive salience to the cues that predicted chocolate milkshake receipt (i.e., sign-track) than those with healthy weight. Additionally, in adolescents, greater caudate and ventral pallidum activity is seen during Pavlovian cue-outcome learning for milkshake compared to water (49, 50) with greater ventral pallidum activity predicting greater increases in BMI 2 years later (49). This finding parallels greater ventral pallidum activity in animal models of sign-tracking (30), suggesting that this may be a common neural pathway for sign-tracking and may be associated with tendency to develop obesity.

The sign-tracking phenotype, in particular, may also play an important role in eating behaviors. While we are not aware of studies examining Pavlovian conditioning, there is one study showing that adults with greater eating restraint were less likely to attribute incentive salience to food cues in a VDAC task (51). This indicates that adults who report a greater tendency to restrict calories are less likely to attribute salience to food cues. There is also a larger literature examining attentional bias to food cues [see reviews (52–54)], which is an indirect measure of incentive salience (27). A recent meta-analysis examining direct [e.g., electroencephalographic (EEG) recordings, eye-tracking] and indirect (e.g., reaction times) measures of food-related attentional bias showed that greater bias was associated with greater hunger, food cravings, and food intake but not body mass index (52). In particular, EEG recordings may be a promising approach for characterizing sign-tracking as late positive event-related potentials (ERPs, e.g., P300 or late positive potentials–LPP) index motivational salience associated with cues (55, 56). In support of this, a recent study used a data-driven approach to cluster adults based on emotional and food-related LLPs with those classified as “sign-trackers” showing larger food-related LLP and higher rates of obesity compared to those classified as “goal-trackers” (57). While late positive ERPs to food-cues is a promising approach for measuring incentive salience and sign-tracking, there is mixed evidence for an association with obesity and binge eating disorder (53). Together, these studies highlight initial evidence that the tendency to attribute incentive salience to food cues (i.e., sign-track) may increase susceptibility to eating behaviors associated with overconsumption [for review of food-cue reactivity beyond incentive salience see (58)].

**FIGURE 2:**
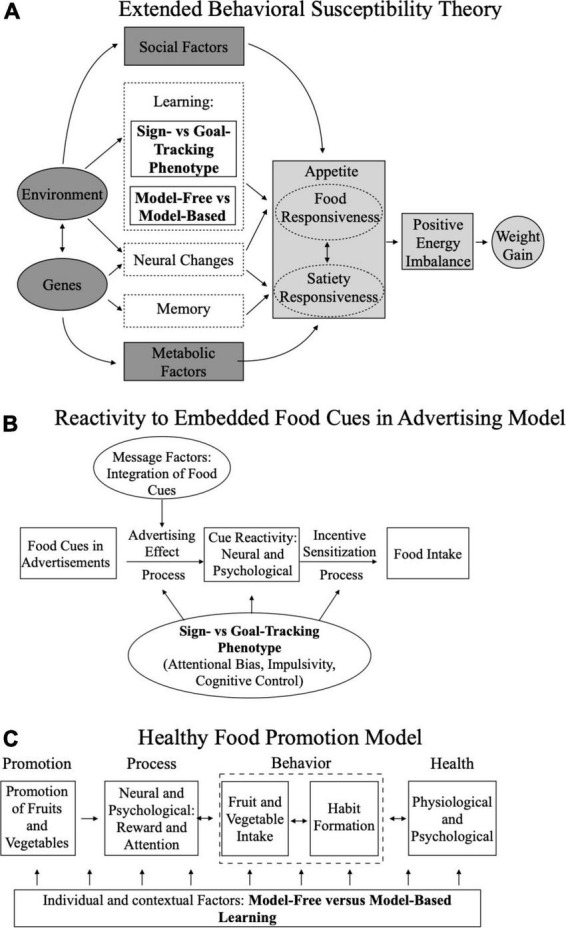
Adapted models with the sign-and goal-tracking and model-free and model-based learning frameworks incorporated. **(A)** Boutelle et al.’s (59) Extended Behavioral Susceptibility; **(B)** Folkvord et al.’s (60) Reactivity to Embedded Food Cues in Advertising Model; **(C)** Folkvord et al.’s (7) Healthy Food Promotion Model.

Based on initial evidence of its role in eating behaviors related to overconsumption and obesity, the sign-tracking phenotype may provide mechanistic insight on the role of food-cue incentive salience in two prevailing models of overconsumption–the Extended Behavioral Susceptibility Theory (59) and the Reactivity to Embedded Food Cues in Advertising Model (REFCAM; Figure 2; 60). The importance of food-cue incentive salience across models highlights its broad potential as a behavioral target for prevention and intervention efforts. For example, cue-exposure therapy aims to reduce food-cue incentive salience by repeatedly exposing participants to a food-cue without the CR of food intake [for review see (12, 61, 62)]. Thus far, cue-exposure therapy has focused on exposures to specific foods, which has been successful in reducing the number of binge eating episodes, number of binge eating days, intake of exposed food, and body weight (63–65) in adults with binge eating disorder and obesity (61–65). While cue-exposure therapy has shown effectiveness for individuals who have already developed food-specific cravings and overconsumption, it is not clear if this approach would be effective for targeting brand or advertising related cues as proposed in the REFCAM model. Additionally, it is not clear if targeting incentive salience would be more efficacious for reducing overconsumption in individuals with sign-tracking compared to goal-tracking phenotypes. Therefore, future work is needed to determine whether targeting individuals based on sign-and goal-tracking phenotypes will contribute to more effective and sustainable weight maintenance.

## Model-free and model-based reinforcement learning

Reinforcement learning is the process through which environmental cues become valued and influence behavior (66). This process is driven by two competing systems–a habitual and a goal-driven system (15, 67–71). The habitual system drives model-free reinforcement learning which relies on stimulus-response (S-R) associations and is a fast, almost automatic, process that requires little cognitive effort (72). For example, stopping for coffee at the same coffee shop on the way to work every day is likely a habitual process. Model-free learning increases the probability of choosing actions that were most recently rewarded, which leads to less accurate and flexible responses. In contrast, the goal-directed system drives model-based learning because it relies on a mental model or cognitive map of the expected value of different responses (i.e., R-O associations) for different “states” or environmental situations. For example, if the coffee shop is closed for maintenance, a goal-directed process is needed to change the morning routine and make coffee at home. Model-based learning leads to more flexible responses; however, it is also more cognitively demanding. These reinforcement learning strategies operate in parallel, with optimal value-based decision-making balancing the need for accuracy with cognitive demand (67, 70, 73, 74).

### Neural pathways that support model-free and model-based learning

Reinforcement learning processes rely on neural encoding of prediction errors, which are used to update outcome expectations and improve accuracy. Model-free learning depends on reward prediction errors (RPEs). A RPE is the difference between the expected outcome and the actual outcome. For example, if someone orders their morning coffee and receives a free donut, that would be a positive RPE. In contrast, if someone orders their morning coffee and receives decaffeinated coffee, that would be a negative RPE. RPEs are encoded by phasic DA signaling in the basal ganglia, which includes ventral striatum, caudate-putamen, and dorsal pallidum (29, 75). In contrast, model-based learning relies on a cognitive model of a task or environmental reward structure so learning is driven by state prediction errors (SPEs). A SPE is the difference between the expected “state” and the actual “state” (70). For example, arriving at coffee shop in the morning and finding it closed for maintenance would be a SPE. SPEs are thought to be encoded by lateral prefrontal cortex, intraparietal sulcus, and anterior cingulate (70, 76, 77). While the neural systems supporting RPEs and SPEs are partially distinct, both model-free and model-based learning include value-based signaling associated with ventral striatal activation (68, 70, 73, 78, 79). A recent meta-analysis showed that in addition to ventral striatum, model-free learning specifically engaged dorsal striatum and dorsal pallidum while model-based learning specifically engaged ventral medial prefrontal cortex and anterior cingulate cortex (79). In addition to regions supporting SPEs, model-based learning also involves dorsolateral prefrontal cortex, orbital frontal cortex, posterior parietal cortex, and hippocampus to support the mental model of different states (15, 71, 80). Given these learning strategies likely operate in parallel (67, 70, 73, 74), common neural correlates for these strategies may help to mediate switching between model-free and model-based learning (15, 69, 81).

### Characterizing model-free and model-based learning

**FIGURE 3:**
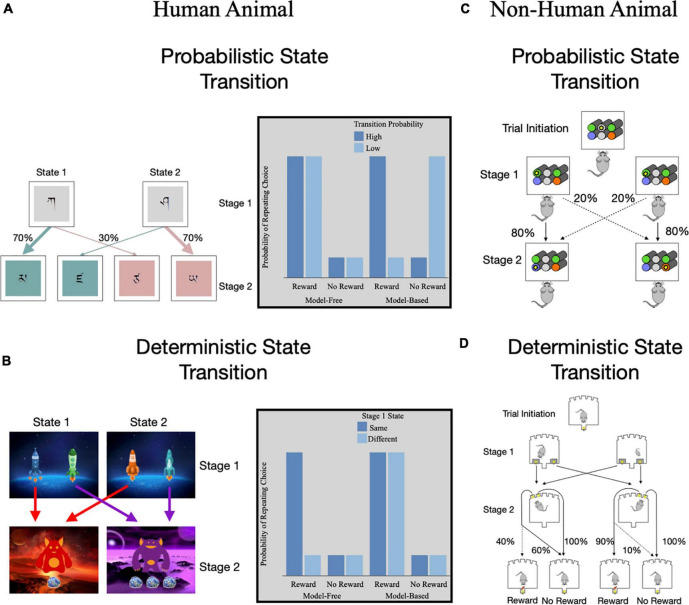
Methods of assessing model-based and model-free reinforcement learning using two-step serial decision-making tasks. Two-step tasks have two stages of decisions with the second stage state dependent upon the first stage choice. **(A)** Daw et al.’s (68) task that uses a probabilistic transition from stage 1 to 2. Gray box highlights the theoretically expected probability of repeating a stage 1 choice for model-free and model-based learning based on previous trial reward and transition probability. **(B)** Kool et al.’s (82) task that uses a deterministic transition from stage 1 to 2. Gray box highlights the theoretically expected probability of repeating a stage 1 choice for model-free and model-based learning based on previous trial reward and whether the current trial stage 1 state differs from the previous trial’s stage 1 state. **(C)** Miller et al.’s (80) translation of a two-stage task for non-human animals with probabilistic state transitions. **(D)** Groman et al.’s (87) translation of a two-stage task for non-human animals with deterministic state transitions.

The advent of computational models for reinforcement learning has propelled our ability to distinguish model-free and model-based learning processes. In particular, the dual-system model incorporates both model-free and model-based algorithms (68, 73) which allows for individual differences in the balance of these systems to be examined. A task structure that leverages the dual-system model is the two-step or serial decision-making task (73, 82). This task involves a series of decisions between two stages. Actions in the first stage lead probabilistically to one of two second-stage states (i.e., high versus low transition probability; Figure 3A). Decisions made in the second-stage then lead to different probabilities of reward, which change or drift slowly throughout the task to encourage learning. The transition structure between stages allows for model-based and model-free strategies to be distinguished. In particular, model-free learners are more likely to repeat an action after a rare or low probability reward due to positive RPE. In contrast, model-based learners will experience a SPE and will be less likely to repeat the action due to the overall low probability of reward. This task has also been adapted to enhance the accuracy-demand tradeoff such that model-based strategies will lead to greater reward (82). In the adapted version, the transitions between the stages are deterministic rather than probabilistic (Figure 3B). Overall, greater use of model-free learning has also been associated with poorer working memory (83, 84), cognitive control (85), and processing speed (86). Therefore, greater reliance on model-free learning during this task is thought to reflect less adaptive reinforcement learning.

While two-step tasks were first developed for human studies, translational applications of the task to rodent models [e.g., (76, 80, 87; Figures 3C,D)] has shown similar patterns of behavior as seen in humans [for reviews on other animal models of habit see (88, 89)]. Animals show evidence of both model-free and model-based learning and evidence for switching between strategies (76, 87, 90, 91). An advantage to animal models is that ability to measure reinforcement learning before and after drug exposure. Drug-naïve animals with less model-free learning exhibited greater subsequent drug administration in animals, while use of model-based learning did not predict subsequent drug administration (92). However, after drug self-administration, rodents showed a reduction in both model-free and model-based learning (92). While this study used a computational model that quantified use of model-free and model-based strategies independently, studies in humans tend to look at the relative use of learning strategies (68, 73) and have shown relatively more model-free than model-based learning in drug users (93). Together, this highlights the importance of having translational assays of decision-making frameworks to better understand behavioral and neural mechanisms of reinforcement learning.

### Relevance to food choice and eating behaviors

While the advent of the dual-system model and two-stage task has led to a swell of research on individual differences in reinforcement learning, little work has directly tested the role of reinforcement learning in food choice and obesity. Of the two studies we are aware of that have directly tested this association one showed greater reliance on model-free learning in adults with obesity compared to those without (94) and one showed no relationship between weight status and reinforcement learning (93). Additionally, model-free learning has been associated with psychological disorders marked by compulsivity including addiction, gambling disorder, obsessive compulsive disorder, and binge eating disorder (93, 95, 96). Model-free learning has been indirectly implicated in overconsumption (97) due to the contribution of compulsivity in habitual overeating (98, 99). Model-free learning may also contribute insight into the Extended Behavioral Susceptibility model, which proposes that “habitual” or instrumental systems contribute to overconsumption (Figures 2A,B; 59). While the majority of the literature and prevailing theories have focused on overconsumption, the Healthy Food Promotion Model proposes that habit learning can be leveraged to bolster intake of fruit and vegetables (Figure 2C; 100). Together, this suggests that interventions that leverage habit learning strategies may be able to increase healthy eating behaviors, but future studies are needed to test this empirically.

## Utility model

Value-based decision-making often involves choosing between multiple actions that could lead to different advantageous outcomes. In utility or value-based decision-making models, every action has an expected value or utility (i.e., action-outcome association) and the action with the highest expected value will be selected. Values associated with different actions are integrated across Pavlovian, instrumental, and goal-directed systems (11) for different consequences of that action, termed attributes. For example, choosing to eat at a restaurant rather than at home could occur because the cumulative value of convenience and taste of food at the restaurant is greater than the value of cost saving and alignment with health goals for eating at home. Thus, the cumulative value of an action integrates both positive and negative value signals across learning systems and attributes. Additionally, the weight of the value signals from different learning systems can be influenced by individual characteristics, such as delay discounting (101, 102). Individuals who value smaller, immediate rewards more than larger, delayed rewards may be more influenced by value signals from the Pavlovian or instrumental systems than the goal-directed system. Further, environmental cues can modulate the weight given to different attributes (e.g., taste, health) (103). For example, an advertisement that draws attention to the palatable aspects of food may increase the value of taste when choosing what to eat. Therefore, value-based decisions are influenced by the subjective value of relevant attributes in addition to self-regulation and environmental contexts.

### Neural pathways that support value-based decision-making

Value-based decision-making relies on the integration of multiple value signals across different learning systems. To compare value signals across dissimilar actions (e.g., take a lunch break or continue reading this paper), a “common currency” or value is encoded in the brain (104, 105). Neuroimaging research suggests that this common value signal is encoded in ventromedial prefrontal cortex and medial orbitofrontal cortex, while value signals for distinct attributes are encoded throughout the brain (106, 107). A meta-analysis showed that when executing reward-based decisions, valuations of different types of reward (e.g., food, money) were associated with activation in ventromedial prefrontal cortex, ventral striatum, posterior cingulate cortex, and superior frontal gyrus; however, only ventromedial prefrontal cortex activity was related to valuations for each reward modality separately (108). This suggests ventromedial prefrontal cortex is a key region for encoding subjective value of both primary rewards like food and secondary rewards like money during decisions. Dorsolateral prefrontal cortex has also been shown to modulate ventromedial prefrontal cortex value signaling during self-control (109, 110) and during context-dependent valuation (111) indicating the importance of both regions in goal-directed decisions. In sum, attribute-specific value signals across the brain are integrated in ventromedial prefrontal cortex, which can be modulated by dorsolateral prefrontal cortex when self-control is engaged or environmental context is important.

### Characterizing value-based decision-making

Characterizing value-based decision-making can involve assessing overall value of an action or stimuli, assessing how different attributes impact overall value, or assessing how psychological and environmental characteristics impact value-based decisions. To estimate the overall expected value of an action, participants can rate how much they want (e.g., strong yes, yes, no, strong no) or how much they are willing to pay for an item (112). Direct ratings of the value of different attributes (e.g., health or taste) have been shown to relate to real world behaviors such as fruit and vegetable intake (113) and smoking initiation (114). These ratings can also be used to examine how attributes influence value-based decision-making by asking participants to make choices between the items. For example, after rating the health and taste of foods, the influence of these attributes on food choice can be examined by having participants choose between food items that differ in taste and health attributes (109, 115–118). Assessing mouse-tracking during these decisions can provide insight into how attributes impact value-based decisions. For example, mouse-tracking trajectories have been used to measure the cognitive effort required to make healthy choices in children (117) and determine when different attributes impact the decision-making process (115, 118). Computational models of decision-making can also be used to examine individual differences in decision-making processes when choosing among options that vary in value. For example, in the Iowa Gambling Task (119) or its adapted child version the Hungry Donkey Task (120), participants try to accumulate as many rewards as possible by repeatedly choosing between four options associated with different reward and punishment probabilities. Computational models can characterize decision-making processes such as how value is updated, consistency between valuation and choice, loss aversion, and sensitivity to the magnitude of gains and losses (121–126). Together, these approaches can be used to understand how individual differences in valuation or cognitive and psychological process relate to disordered or dysregulated eating behaviors.

### Relevance to food choice and eating behaviors

Food choice and eating behaviors require the evaluation of multiple food-related attributes (e.g., taste, health) in addition to personal goals and environmental cues. Taste and health ratings are predictive of food choices in adults (127, 128), however, the impact of these attributes on decisions varies among individuals (109, 128) and can be altered following exposure to taste and health cues (103). These behavioral differences are underpinned by differences in ventromedial and dorsolateral prefrontal cortex activation during decisions (103, 109). In children, taste is more predictive of food choices than health ratings (129, 130), although the temporal dynamics of taste and health attributes on children’s food choices vary by children’s hunger and weight status (115). Additionally, children’s food choices have been shown to be influenced by what they believe their mothers would choose for them (130). For both children and adults, food choices are impacted by many attributes including expectations about the likelihood of feeling satisfied and happy, feeling in control of one’s behavior, eliminating hunger, cost, and convenience (113, 128, 131). This suggests that in addition to food-related attributes, social context, and individual characteristics (132) influence value-based food choices. Understanding the individual characteristics and environmental contexts that influence the value of certain eating behaviors could contribute to interventions that increase the value and selection of foods that optimize health.

Value-based decision-making models complement the other models discussed in this review (Figure 2). For example, a value-based perspective of the Extended Behavioral Susceptibility Theory would suggest that social and environmental factors, genes, and metabolic signals increase the valuation of food cues (i.e., food responsiveness) relative to satiety signals (i.e., satiety responsiveness), contributing to a positive energy balance. Similarly, a value-based perspective of REFCAM would be that food advertisements subconsciously increase the value of food through incentive sensitization, which increases the likelihood of consumption. Correspondingly, interventions that modulate value from social and environmental attributes could lead to changes in food intake. This may include techniques such as cognitive reappraisal and food cue-exposure, which could reduce the value of food cues and increase the relative influence of goal-directed values on food choice. Additionally, manipulations that increase the self-relevancy of goals or influence delay discounting for food may have the potential to influence eating behaviors through their impact on valuation (101). Future research should assess ways to modify food-related value signals across learning systems and attributes and identify who would benefit most from these interventions.

## Discussion

This paper presented three neurocognitive frameworks that could help to advance our understanding of the neurocognitive processes that underly food choices, a critical step toward the development of effective, tailored health interventions. These frameworks support and may help provide mechanistic insight to prominent models for food choice and overconsumption such as the Extended Behavioral Susceptibility model, REFCAM, and the Healthy Food Promotion Model. The sign-and goal-tracking framework can help to provide insight in behavioral phenotypes that may be more susceptible to the attribution of incentive salience to food cues, which could increase craving and overconsumption. The model-free versus model-based framework provides computational models that could be used to better understand habitual intake and compulsive overeating. Finally, the utility or value-based decision-making model provides a framework for understanding how value signals from all three learning systems could be integrated to influence food choice.

The primary advantage of utilizing neurocognitive frameworks is the ability to directly probe valuation and reinforcement learning processes that drive food choice and overconsumption. As the frameworks presented here involve but distinct reward-learning processes, it is often not possible to distinguish causal mechanisms without task behavior. For example, while obesity (133–136), future weight gain (49, 137, 138), and greater food intake (139–141) have all been associated with greater food-cue reactivity in ventral striatum [see (58) for review on neural food-cue reactivity], the interpretation of these findings may differ based on which framework is referenced. Under the sign-and goal-tracking framework, this pattern of results could be interpreted as evidence that greater attribution of incentive salience to food cues drives obesity and overconsumption. In contrast, under the model-free and model-based learning framework, this pattern of results would not be sufficient to make a distinction as both strategies engage ventral striatum (79). However, when considered along with consistent evidence that greater prefrontal cortex engagement is associated with healthy weight (133–136) and lower food intake (142, 143), the combined pattern of results may be interpreted as evidence that greater reliance on model-based strategies is associated with lower weight status and food intake. Alternatively, when using the utility or value-based decision-making framework, the combined pattern of findings could be interpreted as evidence that greater relative value for goal-directed than hedonic values when viewing food cues is protective from excess consumption and adiposity. Therefore, future studies need to assess both neural food-cue reactivity and reward learning. In order to determine how these frameworks mechanistically contribute to different aspects of food choice and overconsumption, ingestive behavior needs to be characterized alongside reward learning and neuroimaging.

All three of these frameworks have utility for better understanding food choice and overconsumption; the choice of which framework(s) to reference ultimately depends on the theory of eating behavior and hypotheses being tested. The sign-and goal-tracking framework enables one to test very specific hypotheses related to the attribution of incentive salience to food cues and its role in motivated behavior such as craving. Model-free and model-based reinforcement learning provides a broader framework to examine reinforcement learning and its role in habitual or compulsive overeating. Lastly, the utility or value-based decision-making theory provides a larger framework to understand how valuation and reinforcement learning processes interact across behavioral control systems during food choice. In sum, applying these frameworks to provide mechanistic insight of prominent models of food choice and overconsumption may eventually contribute to more informed prevention and treatment efforts.

## Author contributions

AP conceptualized the topic and structure and lead the writing and editing of manuscript. BF and KK helped to write and edit the manuscript. All authors contributed to the article and approved the submitted version.
